# The Role of Torsin AAA+ Proteins in Preserving Nuclear Envelope Integrity and Safeguarding Against Disease

**DOI:** 10.3390/biom10030468

**Published:** 2020-03-19

**Authors:** Anthony J. Rampello, Sarah M. Prophet, Christian Schlieker

**Affiliations:** 1 Department of Molecular Biophysics & Biochemistry, Yale University, New Haven, CT 06520, USA; anthony.rampello@yale.edu (A.J.R.); sarah.prophet@yale.edu (S.M.P.); 2 Department of Cell Biology, Yale School of Medicine, New Haven, CT 06520, USA

**Keywords:** TorsinA, nuclear pore complex (NPC), AAA+ ATPase, low-density lipoprotein (VLDL), lipin

## Abstract

Torsin ATPases are members of the AAA+ (ATPases associated with various cellular activities) superfamily of proteins, which participate in essential cellular processes. While AAA+ proteins are ubiquitously expressed and demonstrate distinct subcellular localizations, Torsins are the only AAA+ to reside within the nuclear envelope (NE) and endoplasmic reticulum (ER) network. Moreover, due to the absence of integral catalytic features, Torsins require the NE- and ER-specific regulatory cofactors, lamina-associated polypeptide 1 (LAP1) and luminal domain like LAP1 (LULL1), to efficiently trigger their atypical mode of ATP hydrolysis. Despite their implication in an ever-growing list of diverse processes, the specific contributions of Torsin/cofactor assemblies in maintaining normal cellular physiology remain largely enigmatic. Resolving gaps in the functional and mechanistic principles of Torsins and their cofactors are of considerable medical importance, as aberrant Torsin behavior is the principal cause of the movement disorder DYT1 early-onset dystonia. In this review, we examine recent findings regarding the phenotypic consequences of compromised Torsin and cofactor activities. In particular, we focus on the molecular features underlying NE defects and the contributions of Torsins to nuclear pore complex biogenesis, as well as the growing implications of Torsins in cellular lipid metabolism. Additionally, we discuss how understanding Torsins may facilitate the study of essential but poorly understood processes at the NE and ER, and aid in the development of therapeutic strategies for dystonia.

## 1. Introduction

Over 20 years ago, researchers identified the mutation responsible for causing a severe neurological disorder called DYT1 (for torsion dystonia gene 1) dystonia, which is characterized by involuntary and prolonged muscle contractions [1]. The mutation was mapped to the coding region of a gene that gave rise to a protein called TorsinA, and results in the deletion of a single glutamate at residue position 302/303 where two glutamates repeat in wild type TorsinA [1]. TorsinA was discovered to share regions of homology with the bacterial Clp/Hsp100 family of heat shock proteins [1,2], which are classic examples of ATPases associated with various cellular activities (AAA+) [3]. Since Torsin ATPases (Torsins) have certain conserved sequence features and are phylogenetically related to Hsp100 proteins, they are also classified within the AAA+ superfamily [3]. Despite understanding that they are important for normal neurological development, much remains to be learned about Torsins’ precise molecular function.

**Figure 1:**
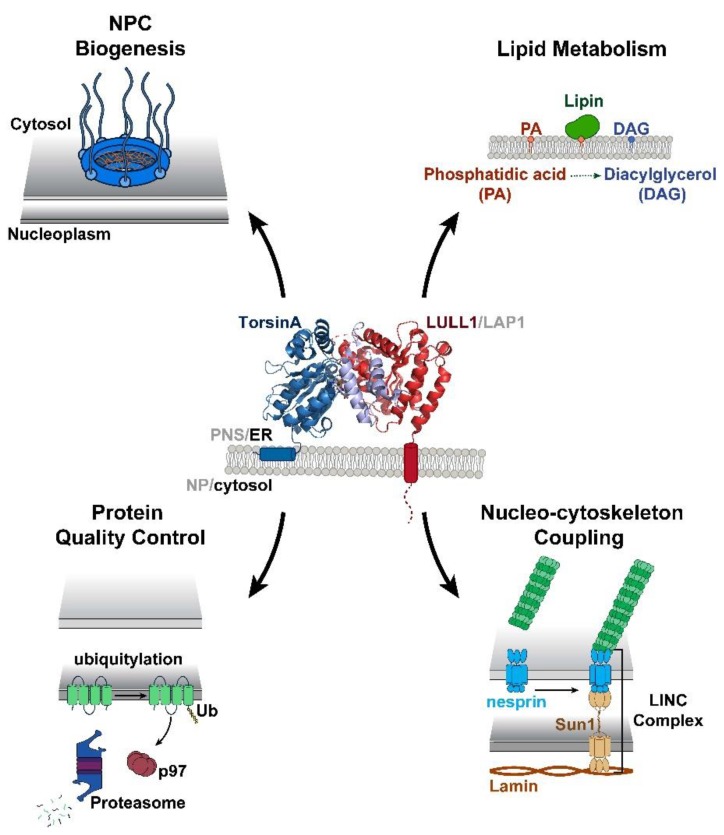
Torsins are implicated in multiple essential cellular processes at the nuclear envelope (NE) and endoplasmic reticulum (ER). The ability of Torsins to participate in these diverse cellular activities relies upon their capacity to efficiently bind and hydrolyze ATP. This ATPase activity is mediated through an active site complementation mechanism in which one of two cofactors contributes a catalytic arginine residue to the active site. Shown is a side view of TorsinA (blue) complexed with the ER-specific cofactor luminal domain like LAP1 (LULL1) (red) (PDB: 5J1S) [12]. The TorsinA RecA fold composed of residues 55–221 is shown in dark blue and the C-terminal α-helical bundle (residues 272–332) is shown in light blue. The LULL1 luminal domain, residues 249–470, is represented in red.

**Figure 2:**
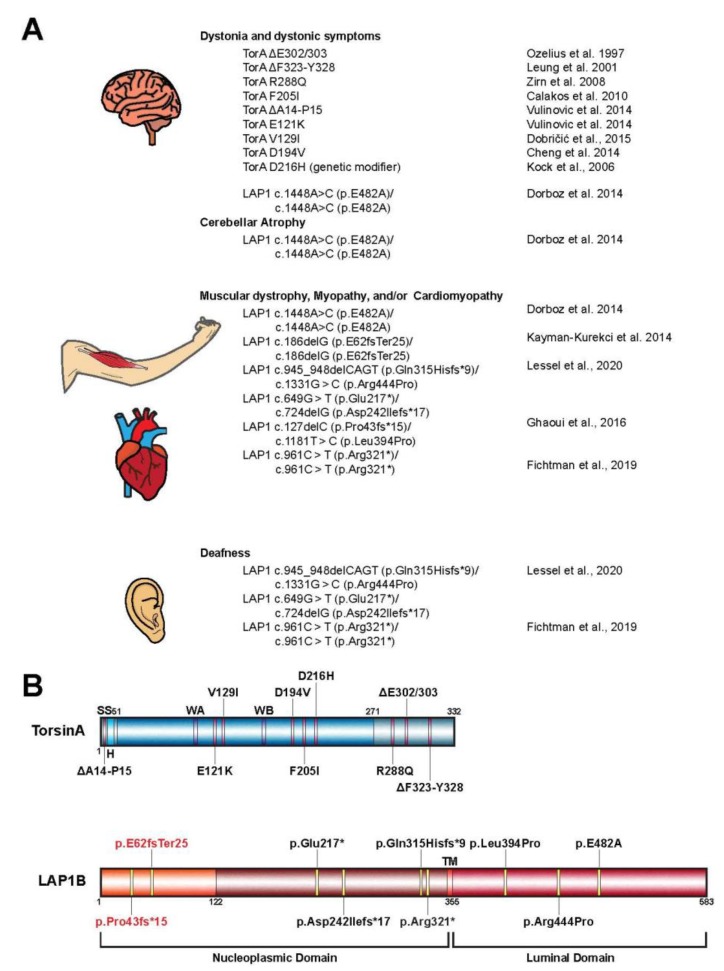
(**A**) Mutations in *TOR1A* and *TOR1AIP1* contribute to a variety of human disease pathologies. Shown is a comprehensive list of disease-associated mutations in the *TOR1A* (TorsinA) and *TOR1AIP1* (lamina-associated polypeptide 1 or LAP1) genes. (**B**) Schematic representation of TorsinA and the LAP1 isoform LAP1B highlighting the alteration associated with the mutations from panel A. LAP1C is a shorter isoform of LAP1 that results from an alternative translation initiation site that ultimately leads to the absence of residues 1–121 [17]. All alterations, except for p.E62fsTer25 and p.Pro43fs*15 (red), are present in both isoforms. SS, signal sequence; H, hydrophobic region; WA, Walker A motif; WB, Walker B motif; TM, transmembrane helix.

Many unique properties of Torsins render them noncanonical AAA+ proteins [4]. For example, they are the only member of the AAA+ superfamily to localize within the endoplasmic reticulum (ER) and nuclear envelope (NE) membrane system [5,6]. One striking deviation from canonical AAA+ proteins is that Torsins lack inherent ATPase activity [7,8]. Instead, Torsins require interactions with one of two transmembrane ATPase-stimulating proteins located within the ER/NE membranes [8] (Figure 1, center). These necessary protein cofactors are lamina-associated polypeptide 1 (LAP1), which localizes to the inner nuclear membrane (INM), and luminal domain like LAP1 (LULL1), which remains within the peripheral ER [5,9]. Through an active site complementation mechanism, LAP1 and LULL1 are individually able to stimulate Torsin’s ATPase activity by donating a catalytic arginine residue to Torsin’s active site that is necessary for transition state stabilization [10,11,12]. The DYT1 dystonia mutation (herein referred to as ΔE) disrupts the TorsinA/cofactor interaction [8,9,10] and consequently, LAP1/LULL1 can no longer stimulate Torsin’s ATPase activity [8,11,12]. Moreover, the ΔE mutation compromises TorsinA homo-oligomerization [8,13]. Further underscoring the importance of the Torsin/cofactor assembly during development is a recent report describing a mutation within the LAP1 gene *TOR1AIP1* that results in a loss of LAP1 expression in patients with dystonia-like symptoms, along with other pathology (see below, Figure 2A,B) [14]. In fact, several disease-associated mutations have been reported for LAP1 (Figure 2A,B) [14,15,16] but none for LULL1, suggesting critical roles for the Torsin/LAP1 complex at the NE [14,15,16].

The human genome encodes four Torsins [1]—TorsinA, TorsinB, Torsin2A, and Torsin3A-that exhibit varying degrees of identity and redundancy. TorsinA remains the best characterized as it is the only one with a known disease-associated mutation. In mammals, TorsinA and TorsinB have a high degree of sequence similarity (~84% in humans) and at least some functional redundancy [18,19,20]. However, their expression patterns are quite different. In many developing neural tissues such as the brain stem and cortex, TorsinA is expressed at a greater level than TorsinB [18,19]. The reverse scenario is true for most non-neural tissues where TorsinB is the dominant isoform [18,19]. Even more distinct is the situation in tissue culture cells, such as mouse embryonic fibroblasts (MEFs) or osteosarcoma (U2OS) cells, where TorsinA and TorsinB are expressed at nearly identical levels [18,19]. These differences may account for the fact that the TorsinAΔE mutant specifically affects neurons.

Understanding the role of Torsins is critical for developing DYT1 dystonia therapies and furthering our knowledge of basic cell biology. However, many distinct properties of Torsins produce a complicated system that has been reported to affect an ever-expanding number of cellular processes (Figure 1). Some of these diverse processes include lipid metabolism [21,22,23], nucleo-cytoskeleton coupling [24,25,26], membrane remodeling [13,27,28], ER redox monitoring [7,29], nuclear pore complex (NPC) biogenesis [30,31,32], and protein quality control [33,34,35,36,37] (Figure 1). In this review, we discuss recent findings that support roles for Torsins in NPC biogenesis and lipid metabolism and speculate how its AAA+ properties may relate to these processes. We also provide an update on Torsins’ oligomeric assembly and biochemistry, and their connection to human health and disease.

## 2. Structural and Biochemical Perspectives on Torsins

AAA+ ATPases are a class of P-loop NTPases with an evolutionarily conserved ATP-binding module that consists of two distinct subdomains: a large wedge-shaped N-terminal α/β RecA fold, and a small C-terminal α-helical domain (Figure 1). In addition to these defining tertiary structural features, all P-loop NTPases are further distinguished by a series of highly conserved amino acid sequence motifs, like the Walker motifs [3,38,39]. Torsins have historically been considered atypical or degenerate AAA+ proteins as they deviate from many of these canonical structural and functional features. Such variations between Torsins and their related ATPases, as well as across the Torsin family, might result from being the sole AAA+ ATPases to reside within the NE and ER, with the demands imposed by these subcellular locations molding their biological activities. Notably, the Walker A motif in Torsins (GXXXXGKN) diverges from the canonical sequence, and has been suggested to partially contribute to their reduced ATPase activity relative to other AAA+ proteins [8,40].

The most striking disparity between Torsins and other AAA+ ATPases, however, is their use of the NE- and ER-specific regulatory cofactors LAP1 and LULL1, respectively, to stimulate their unique mode of catalysis. Alone, Torsins lack the ability to hydrolyze ATP, a peculiarity that renders Torsin distinct from otherwise related Clp/Hsp100 proteins. An in vitro reconstitution of Torsin/cofactor assemblies made the Torsin system amenable to biochemical analysis, which demonstrated that cofactors are stimulators of Torsins’ ATPase activity [8]. Subsequent studies have since shown that ATP hydrolysis is achieved through an active site complementation mechanism in which either cofactor contributes a catalytic arginine residue reminiscent of an arginine finger [41], which is otherwise absent from all known members of the Torsin family [7,11,12,42].

Unlike its related Clp/Hsp100 chaperones, Torsins also lack conserved aromatic-hydrophobic pore loops [12,42] that commonly line the central channel of oligomeric AAA-ATPase ring assemblies and participate in substrate engagement and translocation [43,44]. This critical structural feature allows Clp/Hsp100 proteins to act as unfoldases or chaperones, or, when coupled to for example, protease modules, result in proteolysis of the substrate [39,45]. Although Torsins have been suggested to participate in numerous cellular activities including quality control and chaperone processes, the lack of pore loops and other AAA+ features present some uncertainty regarding their precise mechanistic roles to these pathways.

## 3. Torsin Assemblies and Dystonia Movement Disorders

When considering the members of the Torsin family of ATPases, significant efforts have been placed in elucidating the biological roles of TorsinA due to its direct clinical significance in the highly debilitating movement disorder DYT1 early-onset dystonia [1]. Phenotypic effects of DYT1 dystonia usually manifest in early adolescence and are typified by sustained muscle contractions and involuntary twisting of individual or multiple muscle groups [1,15]. The most penetrant form of this disorder results from the autosomal dominant inheritance of a *TOR1A* variant bearing an in-frame deletion [1]. This mutation leads to the omission of a single glutamate residue in the C-terminal α-helical domain of the encoded protein, termed TorsinAΔE (Figure 2A,B) [12]. While the precise link between TorsinA dysfunction and DYT1 etiology remains unclear, considerable strides have been made over the past decade to further our understanding of the potential molecular and cellular pathways compromised in dystonia patients.

Although Torsins are expressed in all mammalian tissues, TorsinA is distinctly enriched in a subset of cells in the nervous system in mice [18,46,47]. Thus, the differential expression pattern of Torsins likely contributes to the tissue-specific manifestation of TorsinAΔE-linked pathology. Indeed, phenotypes similar to DYT1 dystonia-specific symptoms were observed in conditional mouse models upon deletion of TorsinA in individual brain regions [47,48,49]. These symptoms manifest as abnormal posturing and dystonia-like twisting motions, as well as neurodegeneration of select regions of the central nervous system. The depletion of TorsinA also correlates with the appearance of aberrant protrusions of the INM into the perinuclear space [46,47]. This defect in NE architecture is observed upon manipulation of TorsinA in other model systems including human, fly, and worm cells, suggesting that the function of Torsins at the NE is evolutionarily conserved [27,30,50,51]. Thus, understanding the molecular and functional implications of Torsins in the contexts of these NE aberrations might therefore provide insight into the disease pathogenesis of DYT1 dystonia, as well as dynamic biological processes at the NE.

From a genetic standpoint, the autosomal-dominant inheritance of DYT1 dystonia has long been considered to result from a dominant negative effect of the mutant *TOR1A*(ΔE) allele [52]. While the discussion of a loss-of-function vs. gain-of-function mechanism is still not fully resolved, a loss-of-function mechanism is consistent with the lack of cofactor-induced ATPase activity due to a failure of TorsinAΔE to productively interact with the cofactors LAP1/LULL1 [8,11,12]. In addition, TorsinA deletion or depletion causes dystonic symptoms even in animal models that do not express TorsinAΔE [47,49,53]. In fact, a mildly beneficial effect of expressing TorsinAΔE relative to a Torsin deletion was observed both in animal models [47,48] and tissue culture models [30], suggesting that TorsinAΔE may act as a hypomorphic allele.

On the other hand, Torsins form dynamic higher-order oligomers [8,13,18,54] that rapidly disassemble upon cofactor binding and ATP hydrolysis [55]. Therefore, the addition of TorsinAΔE on the terminal position of TorsinA assemblies could interfere with the disassembly process thereby disrupting the equilibrium between Torsin oligomeric states and ultimately inhibiting their endogenous cellular activities. Moreover, the integration of TorsinAΔE into growing oligomers would represent a defective structure that could interfere with the formation of higher-order oligomers. However, all of these scenarios would require TorsinAΔE to bind to wild type TorsinA in a somewhat stable fashion, which is a questionable scenario given the dynamic instability of the Torsin system [55]. Accordingly, establishing the molecular interactions and functional mechanisms of Torsin/cofactor assemblies is critical for understanding DYT1 dystonia biology, as well as for the development of therapeutic strategies.

Additional mutant alleles of *TOR1A* in patients with varying phenotypic severities have been reported (Figure 2A,B) [56,57,58,59,60,61,62,63,64,65,66]. Importantly, many of these mutations map to regions on TorsinA at the inter-subunit interface, suggesting they perturb Torsin/Torsin or Torsin/cofactor binding [4,12,67]. Supporting the idea that interrupting the Torsin/cofactor interaction is detrimental are reports of patients with mutations in the LAP1 gene, *TOR1AIP1*, who display dystonic-like symptoms [15,68], cardiomyopathy [14,15,68,69], deafness [14,68], and muscular dystrophy [16,69] (Figure 2A). Although many of these phenotypes arising from the *TOR1AIP1* mutation are distinct from those observed in patients with *TOR1A* mutations, a subset of *TOR1AIP1* patients experience dystonic symptoms similar to those presented by DYT1 dystonia (*TOR1A* mutation) patients. Along with the fact that no disease-causing mutations for LULL1 have been reported to date, these observations suggest that LAP1 and Torsins have independent molecular functions in addition to their obviously related contributions to cellular homeostasis.

Though DYT1 dystonia results from the autosomal dominant inheritance of the TorsinAΔE-causing mutation, only ~30% of individuals with the mutant allele exhibit clinical features of the disease [70]. This reduced penetrance is difficult to rationalize and may suggest that additional biological or environmental risk factors contribute to the disease incidence [59,71].

## 4. Emerging Intersections between Torsins and Lipid Metabolism

Since the best-characterized connection between Torsins and human health manifests as a neurological disorder, many Torsin studies have focused on the effects of perturbing the system in neurons (for a review, see [59]). However, Torsins exhibit a broad expression pattern that includes many, if not most, non-neural tissues [18,19]. This feature of the Torsin system, along with its strict conservation even in the lowest extant metazoans [4,42], suggests that these proteins are not strictly limited to participating in neural processes.

One ubiquitous process that may be affected by the Torsin system is lipid metabolism. In *Drosophila melanogaster*, a single, widely expressed Torsin homolog termed *dTorsin* has been suggested to be important for normal adipose tissue development [21]. One study found that *dTorsin* expression within the larval fat body, which is similar to the mammalian liver, was required for viability in flies that are otherwise totally devoid of *dTorsin* [21]. Fly larvae lacking *dTorsin* failed to develop normal fat bodies and exhibited an irregular nuclear exclusion of the phosphatidylcholine biosynthetic enzyme CTP:phosphocholine cytidylyltransferase (CCT) [21], which translocates from the nucleoplasm to the INM upon activation [72,73]. Additionally, these *dTorsin*-null larval fat bodies displayed lower steady state levels of the phosphatidic acid phosphatase lipin but a higher lipin nuclear/cytosolic ratio than wild type fat bodies [21]. Lipin degradation [74] and nuclear translocation [75] have both been reported to occur upon lipin dephosphorylation, which is necessary for lipin to associate with membranes where it carries out its phosphatase activity [76].

The observed nuclear exclusion of CCT in *dTorsin*-null larvae [21] suggests that this enzyme is not activated to the same extent as in wild type animals. Significant hypo-activation of CCT would likely result in decreased bulk membrane biosynthesis within the ER membrane system as CCT’s catalyzed step is rate limiting for phosphatidylcholine production [77], the major structural lipid in eukaryotic membranes [78]. The level of phosphatidylcholine, however, is unperturbed in *dTorsin* knockout (KO) larvae fat bodies [21]. This is consistent with a lipidomic study conducted in HeLa cells devoid of all four human Torsin proteins, which showed no detectable difference in phosphatidylcholine levels in Torsin-deficient cells compared to wild type [30]. Thus, the reason for CCT’s nuclear exclusion upon *dTorsin* manipulation remains to be understood.

Despite the level of phosphatidylcholine in larval fat bodies remaining unaffected by *dTorsin* KO, the lipidomic profiles showed an increase in diacylglycerides (DAG) and a decrease in phosphatidic acid (PtdA) in *dTorsin* KO fat bodies [21]. PtdA is a metabolic intermediate that is converted to DAG by phosphatidic acid phosphatases (PAPs) such as lipin [79,80]. Since lipin was observed to accumulate in the nucleus and presumably degraded in *dTorsin* KO larval fat bodies, both of which are consequences of lipin dephosphorylation and thus its activity-relevant membrane association [74,75], the authors propose that the changes in PdtA and DAG could result from increased lipin activity [21]. A more recent study from the same group reported a direct measurement of lipin1 activity in mouse neural tissue from animals homozygous for the TorsinAΔE mutation [22]. The authors found that lipin1 activity was elevated compared to wild type neural tissue, however, the amount of lipin1 transcript was also increased in the TorsinAΔE +/+ brains compared to wild type [22]. Whether this increase in lipin1 transcript can account for the elevated activity remains to be established. A quantification of steady state lipin1 in mouse neuronal tissue would ultimately be required to compare the lipin degradation observed in *Drosophila* larvae devoid of *dTorsin* [21] with the situation in mice [22].

Future investigation could address the possible functional connection between lipin regulation or turnover and the Torsin system by determining whether the changes in PdtA and DAG can be completely explained by lipin activity or if another PAP could also be contributing. While the aforementioned lipidomics study in HeLa cells [30] did not find any differences in the bulk lipid composition between wild type and Torsin-deficient cells, this analysis would likely have failed to capture local changes at the INM. Therefore, it would be informative to employ lipid probes in Torsin deficient models to detect ER/NE-specific membrane composition changes, which may be relatively transient, and determine what features of the Torsin system contribute to these dynamics. Ultimately, analogous lipidomic analyses need to be performed in animal models due to limitations of tissue cultures models. For example, HeLa cells are notoriously aneuploid and typically cultured in media containing highly variable concentrations of exogenous lipids, a situation that does not necessarily capture physiological homeostatic mechanisms.

An exciting recent report unexpectedly described a role for TorsinA in mouse non-neural tissue that is independent of TorsinB. When mouse livers are conditionally depleted of TorsinA or LAP1, hepatocytes exhibit significant retention of triglycerides and cholesterol [23]. This effect is accompanied by a decrease in the triglyceride secretion rate and plasma cholesterol concentration [23]. The retention of lipids can occur from disrupting any of the three major processes that make up triglyceride metabolism. These processes are lipid breakdown to produce energy, de novo synthesis or uptake of circulating fatty acids, and storage or delivery of fatty acids to peripheral tissue [23]. Fatty acids are stored intracellularly within lipid droplets and transferred to different cells in particles called very low-density lipoproteins (VLDLs), which require apolipoprotein B100 (apoB100) for biogenesis and secretion [81]. Notably, mouse livers depleted of LAP1 are able to normally break down and synthesize lipids, with the amount of circulating fatty acids delivered to the livers remaining the same as in wild type mice [23]. These data imply that the observed defects in lipid metabolism stem from inefficient VLDL secretion. In agreement with this possibility, mouse livers lacking LAP1 or TorsinA secrete significantly less apoB100 [23]. It is important to note that interfering with TorsinA or LAP1 in mouse liver does not produce a global secretion defect, but specifically affects VLDL and apoB100 secretion [23]. Additionally, primary hepatocytes from these knockout livers harbor far less newly synthesized apoB100 at steady state compared to their wild type counterparts [23]. These data demonstrate that the TorsinA-LAP1 complex at the INM plays a major role in VLDL secretion and lipid metabolism in mice—a role that is seemingly independent of TorsinB [82].

Given the data outlined above, developing a unifying model that connects Torsins to lipid metabolism is not straightforward. While mouse liver [23] and fly fat bodies [21] are both reported to accumulate triglycerides during a developmental window upon Torsin manipulation, mouse brains do not [22]. Despite not accumulating triglycerides, mouse brains devoid of TorsinA may exhibit overactive lipin [22] similar to the effect observed in *dTorsin*-KO fly fat bodies [21]. It should be noted, however, that a direct measure of lipin activity has not yet been reported for *dTorsin*-KO fly fat bodies. Notably, lipin activity was not found to be overactive in TorsinA-KO mouse liver [22] where apoB100 production/secretion appears to be compromised and triglycerides retained [23]. Thus, whether lipin is overactive in Torsin-deficient tissue and how this affects triglyceride metabolism remains to be understood, particularly in the hepatic setting where VLDL secretion is dramatically altered upon Torsin manipulation [23].

Since Torsins are present with their activity-stimulating protein cofactors in all metazoans [4,42], their conserved ATPase activity must play an important role for their function. What might this role be? One speculation in the setting of lipid metabolism is that Torsin oligomers act as chaperones within the ER for client proteins such as newly synthesized apoB100 [82]. Since Torsin cofactors have been shown to disassemble Torsin oligomers upon ATP hydrolysis [55,83], the Torsin assembly may release apoB100 into the ER/perinuclear space via LAP1- or LULL1-induced de-oligomerization and consequently substrate release during VLDL maturation.

## 5. The Role of Torsin ATPases in Nuclear Pore Biogenesis

**Figure 3:**
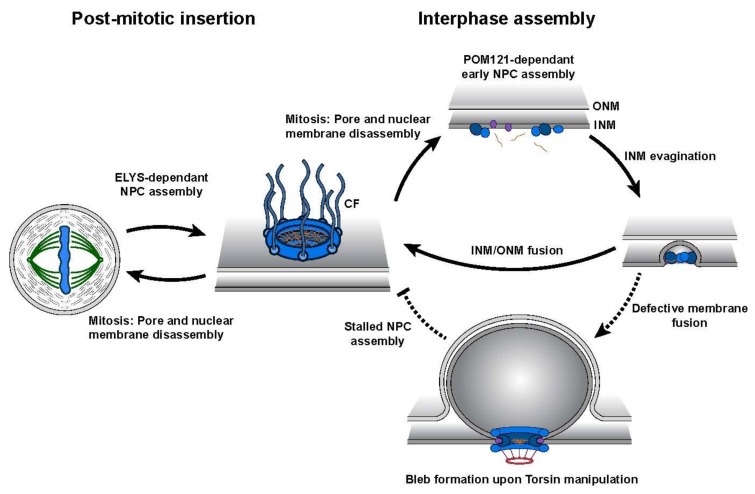
Schematic representation of nuclear pore complex (NPC) biogenesis highlighting the two distinct assembly mechanisms. Post-mitotic insertion occurs during late mitosis where pre-existing NPC subcomplexes assemble at the reforming NE. Interphase assembly requires the de novo construction of NPCs into the double lipid bilayer of the NE. Upon Torsin manipulation, interphase assembly is stalled presumably prior to the fusion of the inner nuclear membrane (INM)/outer nuclear membrane (ONM). Note that cytoplasmic fibrils (CFs) containing NUP358 are only added to the nascent NPC after INM/ONM fusion during interphase assembly (i.e., the observed absence of NUP358 from blebs containing NPC components supports the idea of blebs representing stalled NPC assembly intermediates) [32,84].

While Torsins display unique tissue-specific expression profiles and varying degrees of cofactor stimulation, their manipulation has been extensively linked to the formation of morphological abnormalities at the NE [27,30,46,50,51]. These structures, termed blebs, manifest as omega-shaped INM evaginations that project into the perinuclear space (Figure 3). Such malformations have been observed in numerous model organisms and cell lines, and are detected at early stages of embryonic development suggesting that Torsins perform a critical, evolutionarily conserved biological function at the NE. Recent studies utilizing a series of individual Torsin- and cofactor-deficient cell lines demonstrate that the genetic ablation of Torsins results in phenotypic traits reminiscent of those exhibited by primary mouse neurons harboring the DYT1-causing mutation [30,46]. A comparison of the blebbing phenotype further shows that blebs penetrant most in the altogether Torsin-deficient cell line compared to cell lines that lack only TorsinA and/or TorsinB [30]. This observation suggests that a functional redundancy exists between Torsin paralogs [30].

Although the precise cellular pathway(s) compromised at the NE upon Torsin manipulation remains unclear, growing evidence supports the role of Torsins in nuclear pore complex (NPC) assembly. Mutation of the TorsinA homolog OOC-5 (abnormal OOCyte formation-5) in *Caenorhabditis elegans* prompts the mislocalization of nucleoporins (Nup) and subsequently impairs nuclear trafficking [51]. Moreover, phenylalanine-glycine repeat nucleoporins (FG-Nups) localize to the ‘neck’ regions of blebs while their luminal contents are enriched in ubiquitylated proteins suggesting that these blebs may represent an aberrant NPC intermediate stalled prior to the fusion of the INMs and outer nuclear membranes (ONM) during NPC assembly [30,67]. Changes in the localization of nuclear transport machinery were also observed in neuronal tissue of mouse models bearing conditional TorsinA alleles upon tissue-specific deletion to bypass perinatal lethality [31]. Cases of NE blebbing are not unique to Torsin-deficient systems, but rather have been observed in a steadily increasing list of genetic backgrounds, namely upon the depletion of Nups and NE lipid regulators (for a more detailed review see [85].)

NPC assembly is a multi-faceted process that requires the convergence of numerous proteins and lipids, along with regulatory and quality control systems that surveil the structural and functional integrity of pores. Such systems are essential to maintain nuclear compartmentalization and the permeability barrier that separates the cellular processes of the nucleoplasm from those of the cytosol. This process must also be temporally regulated in order to accommodate the changing growth and energetic demands present at the different phases of the cell cycle. Specialized surveillance systems have evolved in both mammalian and yeast cells that monitor the membrane integrity of the NE, and upon nuclear deformation, activate response systems like the endosomal-sorting complexes required for transport (ESCRT) pathway. In both types of organisms, ESCRT-dependent repair of nuclear rupture events or lesions occur in a process likened to NE reassembly after mitosis, even utilizing some of the same factors (i.e., CHMP7) [86,87,88]. An additional function of the ESCRT system in NPC quality control has emerged in yeast where the recognition of defective pore assemblies or premature fusion of the nuclear membranes is mediated through the integral INM proteins Heh1 and Heh2p [89,90]. ESCRT machinery is then recruited to the defective NPC to re-seal the aberration. While it remains uncertain whether a similar NPC surveillance mechanism exists in higher eukaryotes, defects in NPC assembly are communicated to the abscission checkpoint during cytokinesis [91], a process that is known to involve ESCRT machinery [92,93].

The perforation of the NE during interphase NPC biogenesis poses an intriguing problem for how the fusion of the INM and ONM is coordinated while maintaining the overall structural integrity and compartmentalization of the nucleus. It is therefore tempting to speculate that the NE blebs observed upon Torsin deletion or manipulation result from defects in membrane fusion or remodeling during NPC biogenesis [30,67,83]. Moreover, it is difficult to rationalize why higher eukaryotes conserved a mechanism for NPC assembly that requires Torsin ATPases when lower eukaryotes lack an identifiable Torsin ortholog. The demand for Torsins in NPC assembly must therefore have emerged out of necessity, potentially to address the challenges of open mitosis in which repeated rounds of NE disassembly lead to the mixing of NE- and ER-specific components that must be repartitioned following mitosis.

## 6. Torsins ATPases Contribute to NPC Assembly during Interphase 

Studying the dynamics and molecular mechanics of NPC biogenesis has historically been challenging as pore assembly is a transient process. It also requires a means of discriminating newly constructed from previously assembled NPCs. In higher eukaryotes, NPC biogenesis displays a cell cycle dependency and likely arises through two fundamentally distinct mechanisms. Accumulating evidence suggests that these assembly events occur during NE reformation in late mitosis and during interphase (Figure 3) [84,94,95,96]. In the latter process, a membrane fusion event must occur in order to insert nascent pores into the double lipid bilayer of the NE. This added requirement presumably accounts for the observed difference between interphase and post-mitotic NPC assembly regarding their durations, quality control demands, and membrane remodeling systems (Figure 3).

Through live cell imaging approaches, interphase assembly has been shown to have slower kinetics than post-mitotic NPC insertion likely due to the de novo synthesis of NPC components [96,97]. Additionally, each mode of assembly requires an alternative order of recruited NPC components. Interphase assembly exhibits a selective dependency on the transmembrane protein POM121 (pore membrane protein of 121 kD) while post-mitotic insertion requires ELYS (for embryonic large molecule derived from yolk sac), implying they utilize distinct assembly mechanisms (Figure 3) [95,98]. In a recent study using correlative electron microscopy, researchers were able to construct a temporal model illustrating the growth of interphase assembly intermediates in the G1 phase [84]. Importantly, interphase assembly occurs via an inside-out evagination of the INM in which intermediates grow laterally and towards the ONM [84].

While there is accumulating evidence indicating that Torsins contribute a critical function in NPC biogenesis, their precise role in this molecular pathway remains largely unknown. The recent finding that the loss of TorsinA and its three paralogs in HeLa cells results in a stalling of interphase assembly provides new insight into this mode of NPC assembly [32]. The formation of Nup-containing NE blebs follows a cell cycle-dependent growth and loss pattern, with an increase in the number during interphase and a substantial reduction following mitosis (Figure 3). The accumulation of interphase blebs containing NPC components corresponds to a concomitant decrease in the number of mature NPCs further strengthening the idea of blebs being stalled NPC intermediates. Notably, bleb formation displays a distinct dependency on the presence of POM121—a key component of interphase assembly [95]—while blebs also exhibit an underrepresentation of the late stage NPC component Nup358 relative to other FG-Nups suggesting that stalling might occur prior to membrane fusion (Figure 3).

The dynamics of bleb formation were also monitored through a live cell imaging platform utilizing lattice light sheet microscopy and a fluorescently tagged derivative of myeloid leukemia factor 2 (MLF2), a protein highly enriched in the bleb lumen [32]. These aberrant structures arise rapidly and are remarkably synchronous during early G1 phase at ~700 s after anaphase onset. This is consistent with previous reports of an initial burst in NPC production within the first hour of G1 phase [96,99,100].

The question remains as to why interphase assembly is disrupted in Torsin-deficient cells prior to membrane fusion. The potential for Torsins to be functionally connected to a fusogenic molecular machine is an attractive concept and has been previously proposed [67,83,101].

## 7. Conclusions

The findings discussed above demonstrate that significant progress has recently been made to determine the cellular roles of the mysterious family of Torsin ATPases. These studies specifically contribute to our understanding of how Torsin dysfunction in neurons causes movement disorders. For example, non-dividing neuronal cells are expected to be particularly susceptible to the accumulation of defects resulting from aberrant interphase NPC biogenesis, as this is likely the primary pathway on which neurons rely on for NPC biogenesis following differentiation. Future research aimed at determining the details of both normal and aberrant NPC biogenesis will allow Torsins’ role to be better characterized. Urgent questions that remain involve identifying the fusogenic machinery responsible for outer and INM fusion and defining the precise mechanism through which Torsins contribute to NPC biogenesis.

Recent studies have also demonstrated that Torsins are not only important in developing neurons, but also in non-neural tissue. That TorsinA manipulation affects VLDL secretion in mouse hepatocytes independently of TorsinB [23] {Shin, 2019 #2057} demonstrates that these two proteins, often considered functionally redundant in neurons, may have distinct roles across tissue types. It will be interesting for future studies to interrogate whether patients with DYT1 dystonia have impaired lipid metabolism, and to uncover the critical differences between TorsinA and TorsinB that allow for TorsinA to have such a drastic effect on apoB100 degradation and VLDL secretion.

Our understanding of Torsin ATPases has recently been advanced by the studies described herein, all of which distinctly implicate Torsins in processes that occur within the NE/ER (Figure 1). Thus, they suggest that defects in such processes contribute to the etiology of DYT1 dystonia and other congenital disorders. We anticipate the Torsin field will uncover many more interesting features of these elusive AAA+ proteins, with important ramifications for our understanding of human pathologies caused by mutations in Torsins and their ATPase activators.
